# Biomarkers of oxidative stress, diet and exercise distinguish soldiers selected and non-selected for special forces training

**DOI:** 10.1007/s11306-023-01998-9

**Published:** 2023-04-11

**Authors:** Jesse A. Stein, Emily K. Farina, J. Philip Karl, Lauren A. Thompson, Joseph J. Knapik, Stefan M. Pasiakos, James P. McClung, Harris R. Lieberman

**Affiliations:** 1 Military Nutrition Division, United States Army Research Institute of Environmental Medicine, 10 General Greene Ave, Bldg. 42, Natick, MA 01760 USA; 2 Oak Ridge Institute for Science and Education, Belcamp, MD USA

**Keywords:** Metabolomics, Resilience, Special operations, Physiological strain

## Abstract

**Introduction:**

The metabolomic profiles of Soldiers entering the U.S. Special Forces Assessment and Selection course (SFAS) have not been evaluated.

**Objectives:**

To compare pre-SFAS blood metabolomes of Soldiers selected during SFAS versus those not selected, and explore the relationships between the metabolome, physical performance, and diet quality.

**Methods:**

Fasted blood samples and food frequency questionnaires were collected from 761 Soldiers prior to entering SFAS to assess metabolomic profiles and diet quality, respectively. Physical performance was assessed throughout SFAS.

**Results:**

Between-group differences (False Discovery Rate < 0.05) in 108 metabolites were detected. Selected candidates had higher levels of compounds within xenobiotic, pentose phosphate, and corticosteroid metabolic pathways, while non-selected candidates had higher levels of compounds potentially indicative of oxidative stress (i.e., sphingomyelins, acylcarnitines, glutathione, amino acids). Multiple compounds higher in non-selected versus selected candidates included: 1-carboxyethylphenylalanine; 4-hydroxy-nonenal-glutathione; α-hydroxyisocaproate; hexanoylcarnitine; sphingomyelin and were associated with lower diet quality and worse physical performance.

**Conclusion:**

Candidates selected during SFAS had higher pre-SFAS levels of circulating metabolites that were associated with resistance to oxidative stress, higher physical performance and higher diet quality. In contrast, non-selected candidates had higher levels of metabolites potentially indicating elevated oxidative stress. These findings indicate that Soldiers who were selected for continued Special Forces training enter the SFAS course with metabolites associated with healthier diets and better physical performance. Additionally, the non-selected candidates had higher levels of metabolites that may indicate elevated oxidative stress, which could result from poor nutrition, non-functional overreaching/overtraining, or incomplete recovery from previous physical activity.

**Supplementary Information:**

The online version contains supplementary material available at 10.1007/s11306-023-01998-9.

## Introduction

Military personnel selected for elite military organizations, such as the United States (U.S.) Army Special Operations Force, exhibit distinct biological, physical, and psychological attributes compared to conventional service members (Eskreis-Winkler et al, 2014; Farina et al., 2019; Teplitzky et al, 1991). The Special Forces Assessment and Selection (SFAS) course is a rigorous 19–20 day assessment that identifies Soldiers possessing the physical and mental attributes for the approximately 1–2 years of extended training required for Special Forces Soldiers. During SFAS, candidates experience a multi-stressor environment with real-world occupational consequences and are provided limited information on upcoming events, which intensifies the stress (Farina et al., 2019; Teplitzky et al, 1991). Overall, half of all candidates fail SFAS. Therefore, SFAS provides a unique model to comprehensively investigate both psychological and physiological markers of success in an extremely stressful environment.

Several investigations indicate higher physical performance, physical fitness, lean body mass, and higher cognitive ability are the best available predictors of successful SFAS selection (Teplitzky, 1991; Beal, 2010, Farina et al., 2021; Zazanis, 1999). Biomarkers, such as cortisol, C-reactive protein (CRP), sex-hormone binding globulin (SHBG), dehydroepiandrosterone (DHEA) measured prior to the selection process may also predict success during SFAS (Farina et al., 2019; Ledford et al., 2020; Morgan et al., 2009; Vaara et al., 2020). However, to the best of our knowledge, metabolomic approaches have not been applied to investigate biomarkers that differentiate candidates who succeed from those who do not during SFAS selection, or in various other multi-stressor occupational settings.

Metabolomics, a characterization of hundreds-to-thousands of small-molecules from small quantities of biological samples, has been used to assess metabolic changes associated with military stressors including severe-energy deficit, anabolic resistance, and exhaustive exercise in extreme environments (Karl et al., 2017; Margolis et al., 2021a, b). It has also been used to characterize elevated oxidative stress from intensified physical training (Nieman et al., 2013, 2014) and altered lipid profiles resulting from sleep deprivation (Davies et al., 2014, Ferrell & Chiang 2015), both are stressors commonly encountered by military personnel. This study was designed to build upon previous investigations by identifying differences in the metabolomic profiles of selected and non-selected candidates prior to entering the selection course (SFAS). The study also aimed to determine the relationships between metabolites associated with selection during SFAS and physical performance and diet quality. We hypothesized that pre-SFAS metabolomic profiles would differ between selected and not selected Special Forces candidates, and that these metabolomic differences would be associated with their physical performance and diet quality.

## Methods

### Participants

Participants (N = 761, age = 25 ± 4 years, height = 178.1 ± 7.5 cm, body mass = 82.5 ± 9.2 kg) were active duty, male U.S. Army Soldiers enrolled in SFAS. The candidates were recruited for the study by informational briefings conducted prior to the start of 12 SFAS courses between 2015–2017. All volunteers provided written informed consent. This study presents metabolomics data collected as part of a larger study that assessed factors correlated with success in the SFAS course (Farina et al., 2019, 2020, 2021). The US Army Research Institute of Environmental Medicine Institutional Review Board (IRB) initially approved the study and the U.S. Army Medical Research and Development Command IRB approved the continuing reviews.

### Metabolomics profiling

One day preceding the start of SFAS, fasted blood samples were collected via median cubital venipuncture between 0430h and 0630h. Whole blood was extracted, shipped on dry ice, and stored at − 80^o^C until being sent to Metabolon, Inc. (Durham, NC) for global metabolomic profiling. Metabolomics analysis proceeded as previously described (Karl et al., 2017; Ford et al., 2020). Briefly, samples were analyzed using four separate methods: two separate reverse-phase (RP)/UPLC-MS/MS methods with positive ion mode electrospray ionization (ESI), a RP/UPLC-MS/MS method with negative ion mode ESI, and a HILIC/UPLC-MS/MS method with negative ion mode ESI. All analysis methods utilized a Waters ACQUITY UPLC (Waters Corp., Milford, MA) and a Thermo Scientific Q-Exactive high-resolution/accurate mass spectrometer interfaced with a heated ESI-II source and Orbitrap mass analyzer operated at 35,000 mass resolution. One aliquot was analyzed using acidic positive ion conditions, chromatographically optimized for more hydrophilic compounds. The extract was gradient eluted from a C18 column (Waters UPLC BEH C18-2.1x100 mm, 1.7 µm) using water and methanol, containing 0.05% perfluoropentanoic acid (PFPA) and 0.1% formic acid (FA). Another aliquot was analyzed using acidic positive ion conditions, chromatographically optimized to detect more hydrophobic compounds, by gradient eluting from the same C18 column using methanol, acetonitrile, water, 0.05% PFPA and 0.01% FA operating at an overall higher organic content. Another aliquot was analyzed using basic negative ion optimized conditions using a separate dedicated C18 column. The basic extracts were gradient eluted from the column using methanol and water with 6.5mM ammonium bicarbonate (pH = 8). The fourth aliquot was analyzed via negative ionization following a hydrophilic interaction liquid chromatography (HILIC) type separation on an amide column (Waters UPLC BEH amide 2.1x150 mm, 1.7 µm). The separation was achieved using a gradient consisting of water and acetonitrile with 10mM ammonium formate (pH = 10.8). All mass spectrometry (MS) analysis alternated between MS and data-dependent multistage mass spectrometry (MSn) scans using dynamic exclusion. The scan range varied slighted between methods but covered 70-1000 mass to charge ratio (m/z). Additional information regarding UPLC-MS methods are provided (Supplement 10).

Technical replicates, blanks, internal standards, and several recovery standards were analyzed with experimental samples for quality control. Raw data were extracted, peak identified, and quality control processed using proprietary hardware and software (Metabolon, Inc.). Identification of peaks was based on comparing retention times, mass to charge ratios, and chromatographic data retained within a library maintained by Metabolon which contains entries of purified standards and recurrent unknown entities. Peaks were quantified as area under the curve, which were used for statistical analyses.

### Diet quality

Usual dietary intake was determined with a 127-item 2014 Block food frequency questionnaire (NutritionQuest, Berkeley, CA). The food frequency questionnaire was administered prior to starting SFAS. Food frequency (number of times a food item was consumed per month or week), and quantity (portion size) was reported for each food item to derive daily consumption of foods, total energy intake, and diet quality as measured by Healthy Eating Index (HEI-2015) scores. HEI-2015 total and individual component scores were calculated according to minimum and maximum score standards described previously (Krebs-Smith et al., 2018). The nine individual HEI-2015 component scores that assess compliance with the 2015 Dietary Guidelines for Americans recommended intakes included: (1) total vegetables; (2) greens and beans; (3) total fruit; (4) whole fruit; (5) whole grains; (6) dairy; (7) total protein foods; (8) seafood and plant protein; and (9) fatty acid ratio (polyunsaturated fatty acids + monounsaturated fatty acids)/saturated fatty acids). Four additional components assessed compliance with recommendations for moderate intake, including (1) sodium, (2) refined grains, (3) added sugar, and (4) saturated fats. The HEI-2015 score ranged from 0 to 100 and was calculated by the sum of all components, with higher scores indicating greater compliance with the Dietary Guidelines for Americans (Krebs-Smith et al., 2018). We previously established that HEI-2015 total score, as well as component scores for total vegetables, greens and beans, seafood and plant protein, sodium, and refined grains, were associated with higher physical performance and higher likelihood of selection in this cohort (Farina et al., 2020). Thus, we focused on these same diet quality outcomes in the present analysis.

### Physical performance and selection outcome

Several physical events/assessments were administered by SFAS instructors as routine requirements during the course. This investigation focused on the Army Physical Fitness Test (APFT), timed runs, timed loaded road marches, and land navigation performance since they had previously been demonstrated to be the most associated with selection in this cohort (Farina et al., 2019). The APFT is a scored physical assessment with a maximum score of 300 that consists of three events, 2-minutes of push-up, 2 minutes of sit-ups, and a 2-mile run (100 possible points for each event). Candidates completed two additional timed runs and timed loaded road marches. The distance of the runs and road marches were the same for each course iteration and performance was assessed by time to event completion. Land navigation performance was determined by total number of grid coordinates (locations) successfully found in the allowable time limit in an unfamiliar wooded terrain using only a paper map and compass.

### Statistical analysis

Batch-normalized data, in which the measured area under the curve (AUC) for each metabolite in the experimental samples, divided by the median AUC of that metabolite within all samples measured in each instrument batch, were provided by Metabolon. Data were then exported into MetaboAnalyst (Version 5.0) for analysis (Pang et al., 2021). Analyzed baseline samples (*N* = 747) containing 1026 compounds (144 or 14% unknown compounds) underwent data processing as previously described (Stein et al., 2022). Metabolic features were removed if not detected in > 20% of samples and any remaining missing data were imputed using 1/5 of the minimum value for that feature across all samples. Non-informative variables (e.g., very small values, nearly constant values, variables with low repeatability) were detected and filtered using interquantile ranges. Data were then log_10_ transformed and auto-scaled (mean-centered and divided by the standard deviation of each variable) prior to analysis.

Principal component analysis (PCA) was used to visualize differences in the metabolomes of selected and non-selected candidates. Mann-Whitney U-tests were used to identify individual compounds that differed between groups. False discovery rates (FDR) were determined for Mann-Whitney U tests by adjusting p-values with a Benjamini-Hochberg correction. Metabolites that were significantly different between selected and non-selected candidates were then used as independent variables in multiple linear regression models with stepwise selection wherein physical performance outcomes served as the dependent variables. Pearson’s correlation analysis was conducted to examine the relationships between metabolites that were statistically significant in multiple physical performance models and diet quality outcomes. Statistical significance was set to α < 0.05 and FDR < 0.05.

## Results

### Metabolomic signatures of Special Forces candidates

Following data processing and filtering, 609 compounds remained for statistical analysis. PCA analysis showed subtle separation between selected versus not selected candidates with PCA components 1 and 2 capturing 11% of the variance in metabolomic signatures between candidates (see PCA Figure, Supplement 1).

**Fig. 1:**
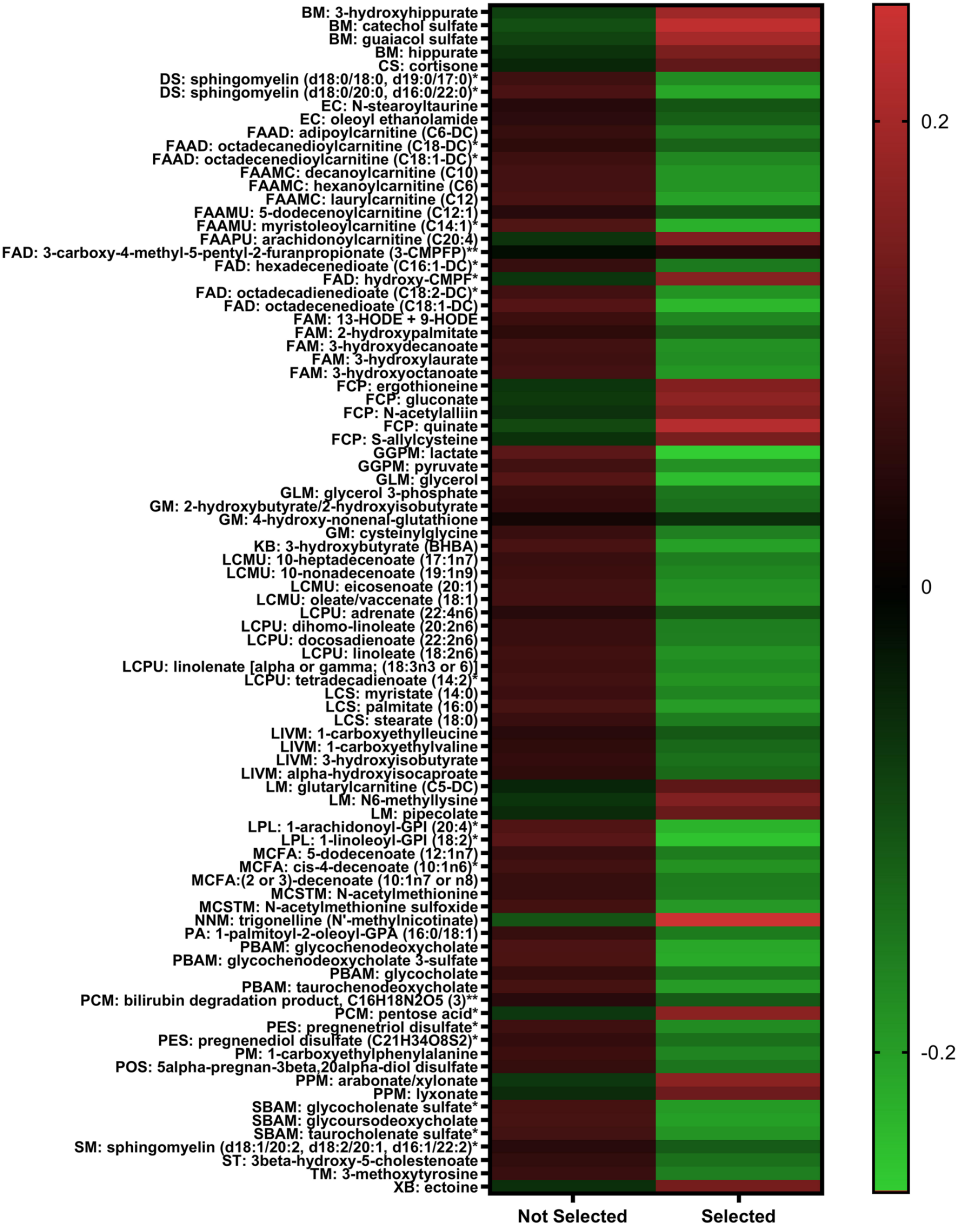
Metabolomic differences between selected and non-selected candidates at pre-SFAS. Values represent median log_10_ auto-scaled AUC. Abbreviations represent metabolic subpathways including benzoate metabolism (BM), corticosteroids (CS), dihydrosphingomyelins (DS), endocannabinoid (EC), fatty acid metabolism - acylcarnitine, dicarboxylate (FAAD), fatty acid metabolism - acylcarnitine, medium chain (FAAMC), fatty acid metabolism - acylcarnitine, monounsaturated (FAAMU), fatty acid metabolism - acylcarnitine, polyunsaturated – (FAAPU), fatty acid – dicarboylate (FAD), fatty acid – monohydroxy (FAM), food component/plant (FCP), glycolysis, gluconeogenesis, and pyruvate metabolism (GGPM), glycerolipid metabolism (GLM), ketone bodies (KB), long chain monounsaturated fatty acid (LCMU), long chain polyunsaturated fatty acid (LCPU), long chain saturated fatty acid (LCS), leucine, isoleucine and valine metabolism (LIVM), lysine metabolism (LM), lysophospholipid (LPL), medium chain fatty acid (MCFA), methionine, cysteine, SAM and taurine metabolism (MCSTM), nicotinate and nicotinamide metabolism (NNM), phosphatidic acid (PA), primary bile acid metabolism (PBAM), partially characterized molecules (PCM), pregnenolone steroids (PES), phenylalanine metabolism (PM), progestin steroids (POS), pentose metabolism (PPM), secondary bile acid metabolism (SBAM), sphingomyelins (SM), sterol (ST), tyrosine metabolism (TM), xenobiotic (XB)

After Benjamini-Hochberg false discovery correction, Mann-Whitney U tests revealed between-group differences (FDR < 0.05) in 108 of the 609 metabolites (17.7%) (Fig. 1) (see Table and Heatmap, Supplement 2–3). Differences between selected and non-selected candidates were primarily observed in compounds within lipid (*n* = 59, 55%), amino acid (*n* = 14, 13%), and xenobiotic (*n* = 10, 9%) metabolism pathways. Statistically significant compounds exhibited similar trends between groups across their respective subpathways. For instance, compounds within leucine, isoleucine and valine (*n* = 4), glutathione (*n* = 3), methionine (*n* = 2), glycolysis (*n* = 2), long chain fatty acids (unsaturated, polyunsaturated, and saturated) (*n* = 13), acylcarnitine (*n* = 9), monohydroxy fatty acid (*n* = 5), dicarboxylate (*n* = 5), and primary (*n* = 4) and secondary bile acid (*n* = 3) metabolic pathways were significantly lower in selected candidates, with the exception of two dicarboxylate fatty acids (e.g., 3-carboxy-4-methyl-5-pentyl-2-furanpropionate, hexadecenedioate) and one acylcarnitine (e.g., arachidonoylcarnitine). Compounds within pentose phosphate (*n* = 3) and benzoate (*n* = 4) metabolic pathways were consistently higher in selected candidates (Fig. 1).

### Metabolomic correlates of physical performance and their relationship to diet quality

**Fig. 2:**
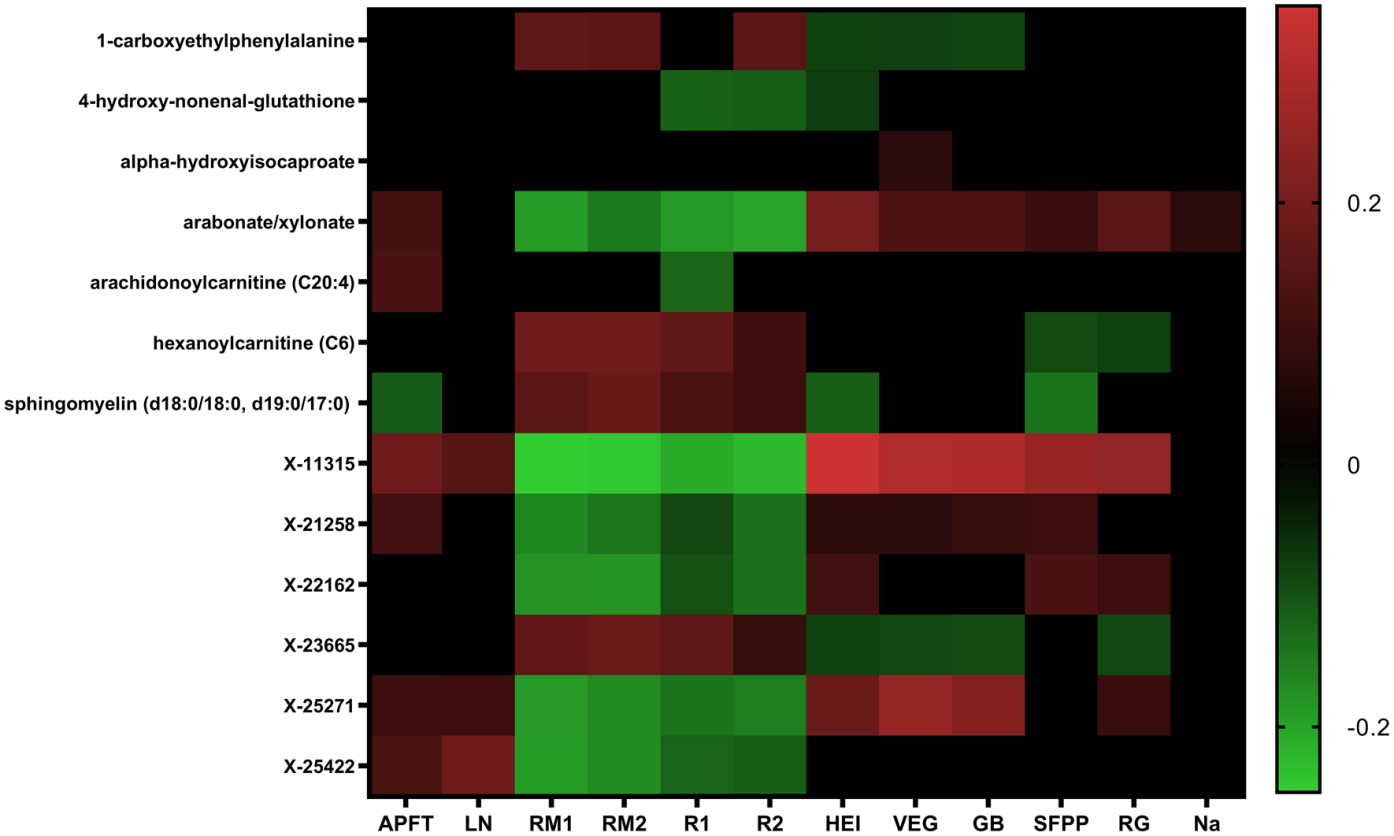
Metabolomic correlations with physical performance and diet quality outcomes. Values represent Pearson’s correlation coefficients. Black indicates non-significant relationships. APFT represent Army Physical Fitness Test, LN represents Land Navigation, RM1 represents Road March 1, RM2 represents Road March 2, R1 represents Run 1, R2 represents Run 2, HEI represents HEI-2015 scores, VEG represents Total Vegetable, GB represents Greens & Beans, SFPP represents Seafood & Plant Protein, RG represents Refined Grains, and Na represents Sodium

Multiple linear regression revealed significant associations between metabolites and all physical performance outcomes (adjusted R^2^ = 0.143 to 0.218, *p* < 0.001) (see Tables, Supplement 4–9). Within these models, 13 metabolites were predictive of > 1 physical performance outcome (Fig. 2). All of these metabolites, except for arachidonoylcarnitine and X-25422, were also correlated with at least one metric of diet quality (Fig. 2). Higher sphingomyelin (d18/18:0, d19:0/17:0), hexanoylcarnitine (C6), 1-carboxyethylphenylalanine, and X-23665 levels were associated with both lower performance on load-carriage road marches and run times and worse diet quality scores. In contrast, higher arabonate/xylonate, X-11315, X-21258, X-25271, and X-25422 were associated with better physical performance on multiple tasks and higher diet quality scores.

## Discussion

This study assessed circulating (blood) metabolomic signatures of US Army Soldiers immediately prior to their entry into the Special Forces Assessment and Selection (SFAS) course. In 18% of detected metabolites, there were significant between group differences between Special Forces candidates who were selected and those not selected for additional Special Forces training. Prior to entering SFAS, non-selected candidates had higher levels of sphingomyelins, acylcarnitines, glutathione, and compounds within branched chain amino acid metabolic pathways than selected candidates. While oxidative stress was not directly measured in this study, several of these compounds can reflect elevated oxidative stress (Karl et al., 2017; Nieman et al., 2013, 2014). Selected candidates had higher levels of circulating xenobiotic and pentose phosphate metabolites, which could indicate higher intake of antioxidant-rich foods and greater capacity to resynthesize glutathione. These findings suggest that the individuals not selected to continue with Special Forces training are characterized by metabolomic profiles indicative of greater oxidative stress before starting SFAS than those who are selected.

Increased oxidative stress in non-selected candidates may reflect intensified exercise training, and/or incomplete recovery from that training prior to entering SFAS. Elevated amino acid, long chain fatty acids, and glutathione metabolites that were higher in non-selected candidates have also been observed in recreational athletes after prolonged exercise and intensified exercise training (Nieman et al., 2013, 2014). Several fatty acids (i.e., palmitate, myristate, linoleate) were elevated in the non-selected SFAS candidates in this study. Oxidized linoleate generates 13-HODE + 9-HODE, a marker of oxidative stress elevated after strenuous exercise (Nieman et al., 2014), and was also elevated in non-selected vs. selected candidates. Collectively, these higher metabolic markers indicative of oxidative stress among non-selected candidates may suggest some degree of nonfunctional overreaching or overtraining had occurred during the days and weeks before entering SFAS.

Glutatione metabolites were elevated in non-selected candidates prior to entering SFAS and also suggests the presense of oxidative stress. Reduced glutathione protects against oxidative stressors by reducing reactive oxygen species and is upregulated during intense military training (Lu, 2013; Karl et al., 2017). The pentose phosphate pathway plays a crucial role in recycling oxidized glutathione. Higher yields of NADPH from this pathway may enhance the capacity to resynthesize reduced glutathione, increasing the resistance to oxidative stress (Powers & Jackson, 2008). This study found that selected candidates had elevated pentose phosphate metabolites, which could indicate a distinct training adaptation or phenotype previously observed in elite high-power athletes (Al-Khelaifi et al., 2018). These findings suggest that pentose phosphate metabolites may serve as biomarkers of physiological resilience that deserve further attention.

Selected Special Forces candidates consumed healthier diets than their non-selected peers, which contain higher levels of xenobiotic compounds, indicating they were choosing unique foods (Farina et al., 2020). Although fruit and whole grain consumption were not different between selected versus non-selected candidates as assessed by food frequency questionnaires (Farina et al., 2020), metabolomics analysis revealed higher levels of benzoate metabolites in selected candidates and suggest otherwise (Nieman et al., 2013; Pallister et al., 2017). N-acetylalliin and S-allylcysteine, in addition to quinate and trigonelline, were also elevated in selected candidates. These compounds are found and fruits, vegetables, allium vegetables, and coffee beans and can act as dietary antioxidants, potentially contributing to the observed differences in oxidative stress metabolites (Wang et al., 2018; Dobrev et al., 2020; Kothari et al., 2020).

Circulating sphingomyelins are inversely related to aerobic fitness and can accumulate under conditions of oxidative stress (Karl et al., 2017; Lustgarten et al., 2013, Nikolova-Karakashian & Reid, 2011). The higher sphingomyelin (d18:0/18:0, d19:0/17:0) levels observed in this study were associated with lower diet quality and slower road march and running evaluation times. Sphingomyelinase hydrolyzes sphingomyelins into ceramide, which can impair muscle function by upregulating sphingosines (Nikolova-Karakashian & Reid., 2011; Powers et al., 2011; Sabbadini et al., 1992). Oxidative stress and poor diet quality may have contributed to elevated sphingomyelin levels and decreased physical readiness in the non-selected candidates before entering SFAS.

Non-selected candidates had higher levels of acylcarnitines and amino acid metabolites. Higher levels of arachidonoylcarnitine were correlated with faster run times, while higher levels of hexanoylcarnitine were associated with slower run times. Acylcarnitines may indicate a mismatch between fatty acid availability and metabolic capacity (Lehmann et al., 2010; Koay et al., 2021), and shorter chain acylcarnitines (e.g., hexanoylcarnine) that do not bind to oxymyoglobin (Chintapalli et al., 2016), predict lower aerobic capacity (Lustgarten et al., 2013). Acylcarnitines can also be formed by catabolism of certain amino acids. For example, 1-carboxyethylphenylalanine, a phenylalanine metabolite, was associated with lower diet quality and slower road march and run times. This metabolite also predicted slower gait speeds in middle-aged adults (Nierenberg et al., 2020). Elevations in 1-carboxyethylphenylalanine might indicate that skeletal muscle-derived amino acids were being used for energy production, or a general catabolic state that resulted from overtraining, poor sleep, oxidative stress, or low diet quality (Cedernaes et al., 2018, Margolis et al., 2021b, Petibois et al, 2002).

To the best of our knowledge, this is the first, large-scale metabolomic investigation of success or failure among healthy individuals completing an arduous occupational selection program. This study does, however, have limitations. This study used data from a fasted sample at a single time point and did not characterize any dietary or physical activity changes leading up to SFAS. Additionally, this study was unable to capture all volunteers’ physical performance outcomes since candidates drop out as they fail to meet requirements during the course or can withdraw from the selection process voluntarily and are not available for this study. While this study employed a comprehensive metabolomics analysis, traditional oxidative stress markers were not measured. Also, while this study’s cross-sectional design found statistically significant associations between metabolites and physical performance and diet quality, those associations were weak and do not establish causation between these outcomes.

## Conclusion

Before beginning the Special Forces Assessment and Selection (SFAS) course metabolomic profiles of Special Forces candidates differed between those ultimately selected for continued Special Forces training versus those not selected. Non-selected candidates exhibited higher levels of metabolic biomarkers indicative of oxidative stress, while selected candidates may possess a greater capacity to resynthesize glutathione to lower oxidative stress via pentose phosphate metabolites. Several of these metabolites were associated with the candidate’s physical performance and diet quality, and suggest that the presence of oxidative stress and/or the ability to mitigate oxidative stress may influence a candidates physical performance and likelihood of selection. Thus, the use of a comprehensive metabolomics approach may provide additional insight into the factors that determine success, and stress resilience in a multi-stressor environment, compared to relying on any single individual biomarker. Future research should aim to validate these findings in other populations, and should also consider other potential biomarkers (i.e., microRNAs) and multiomics biomarkers.

## Supplementary Information

Below is the link to the electronic supplementary material.

Supplementary material 1 (DOCX 134.4 kb)

Supplementary material 2 (DOCX 19.8 kb)

Supplementary material 3 (DOCX 20.2 kb)

Supplementary material 4 (DOCX 20.1 kb)

Supplementary material 5 (DOCX 20.9 kb)

Supplementary material 6 (DOCX 36.0 kb)

Supplementary material 7 (DOCX 125.1 kb)

Supplementary material 8 (DOCX 20.5 kb)

Supplementary material 9 (DOCX 20.5 kb)

Supplementary material 10 (DOCX 18.7 kb)

## Data Availability

Supplementary data available. Database cannot be made publicly available due to agreements with United States Special Operations Command (USSOCOM).
