# Supplementary material for: Biomarkers of oxidative stress, diet and exercise distinguish soldiers selected and non-selected for special forces training
Source: Metabolomics. 2023 Apr 11;19(4):39. doi: 10.1007/s11306-023-01998-9 (PMC10090007; doi:10.1007/s11306-023-01998-9)

## Supplement 1: Principal component analysis of candidate metabolomics data. A score plot of the first (PC1) and second principal components (PC2) indicating similar clustering between selected and non-selected candidates.


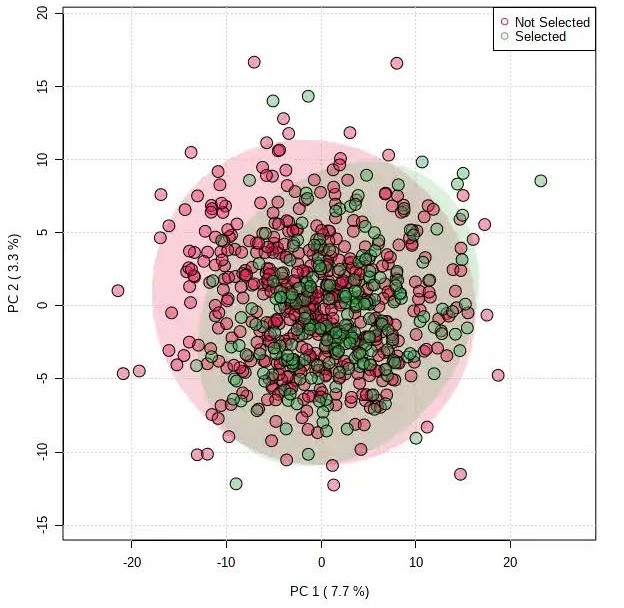

Supplement: Supplementary file 1 — Supplementary material 1 (DOCX 134.4 kb) [file 11306_2023_1998_MOESM1_ESM.docx]
