# Supplementary material for: Biomarkers of oxidative stress, diet and exercise distinguish soldiers selected and non-selected for special forces training
Source: Metabolomics. 2023 Apr 11;19(4):39. doi: 10.1007/s11306-023-01998-9 (PMC10090007; doi:10.1007/s11306-023-01998-9)
Supplement: Supplementary file 2 — Supplementary material 2 (DOCX 19.8 kb) [file 11306_2023_1998_MOESM2_ESM.docx]

## Supplemental Digital Content 9: Multiple Linear Regression Coefficients for Run 2

| **Subpathway** | **Name** | **β** | **t** | ***p*** |
| --- | --- | --- | --- | --- |
|  | (Constant) |  | 321.873 | 0.000 |
| Unknown metabolite | X-11315 | -0.139 | -3.525 | 0.000 |
| Pentose Metabolism | arabonate/xylonate | -0.121 | -3.140 | 0.002 |
| Glycolysis, Gluconeogenesis, and Pyruvate Metabolism | lactate | 0.092 | 2.076 | 0.038 |
| Glutathione Metabolism | 4-hydroxy-nonenal-glutathione | -0.152 | -4.077 | 0.000 |
| Unknown metabolite | X-21258 | -0.112 | -2.987 | 0.003 |
| Unknown metabolite | X-25271 | -0.110 | -2.834 | 0.005 |
| Phenylalanine Metabolism | 1-carboxyethylphenylalanine | 0.108 | 2.429 | 0.015 |
| Benzoate Metabolism | catechol sulfate | -0.096 | -2.416 | 0.016 |

Adjusted R^2^ = 0.143, p < 0.001.
