# Supplementary material for: Biomarkers of oxidative stress, diet and exercise distinguish soldiers selected and non-selected for special forces training
Source: Metabolomics. 2023 Apr 11;19(4):39. doi: 10.1007/s11306-023-01998-9 (PMC10090007; doi:10.1007/s11306-023-01998-9)
Supplement: Supplementary file 3 — Supplementary material 3 (DOCX 20.2 kb) [file 11306_2023_1998_MOESM3_ESM.docx]

## Supplement Digital Content 6: Multiple Linear Regression Coefficients for Road March 1

| **Subpathway** | **Name** | **β** | **t** | ***p*** |
| --- | --- | --- | --- | --- |
|  | (Constant) |  | 223.892 | 0.000 |
| Unknown metabolite | X-11315 | -0.143 | -3.875 | 0.000 |
| Secondary Bile Acid Metabolism | taurocholenate sulfate* | 0.123 | 3.339 | 0.001 |
| Unknown metabolite | X-25422 | -0.124 | -3.437 | 0.001 |
| Phenylalanine Metabolism | 1-carboxyethylphenylalanine | 0.176 | 4.964 | 0.000 |
| Unknown metabolite | X-21258 | -0.126 | -3.561 | 0.000 |
| Unknown metabolite | X-23665 | 0.105 | 2.876 | 0.004 |
| Unknown metabolite | X-25271 | -0.087 | -2.342 | 0.020 |
| Corticosteroids | Cortisone | -0.096 | -2.718 | 0.007 |
| Pentose Metabolism | arabonate/xylonate | -0.098 | -2.688 | 0.007 |
| Fatty Acid Metabolism (Acylcarnitine) | hexanoylcarnitine (C6) | 0.097 | 2.626 | 0.009 |
| Dihydrosphingomyelins | sphingomyelin (d18:0/18:0, d19:0/17:0)* | 0.080 | 2.219 | 0.027 |

Adjusted R^2^ = 0.218, p < 0.001.
