# Supplementary material for: Biomarkers of oxidative stress, diet and exercise distinguish soldiers selected and non-selected for special forces training
Source: Metabolomics. 2023 Apr 11;19(4):39. doi: 10.1007/s11306-023-01998-9 (PMC10090007; doi:10.1007/s11306-023-01998-9)
Supplement: Supplementary file 4 — Supplementary material 4 (DOCX 20.1 kb) [file 11306_2023_1998_MOESM4_ESM.docx]

## Supplement Digital Content 5: Multiple Linear Regression Coefficients for Land Navigation

| **Subpathway** | **Name** | **β** | **t** | ***p*** |
| --- | --- | --- | --- | --- |
|  | (Constant) |  | 65.253 | 0.000 |
| Unknown metabolite | X-25422 | 0.191 | 4.219 | 0.000 |
| Unknown metabolite | X-16935 | -0.135 | -2.924 | 0.004 |
| Leucine, Isoleucine and Valine Metabolism | alpha-hydroxyisocaproate | -0.152 | -3.384 | 0.001 |
| Glycolysis, Gluconeogenesis, and Pyruvate Metabolism | Pyruvate | -0.109 | -2.391 | 0.017 |
| Unknown metabolite | X-18913 | 0.120 | 2.480 | 0.014 |
| Fatty Acid Metabolism(Acylcarnitine) | adipoylcarnitine (C6-DC) | -0.137 | -2.893 | 0.004 |
| Fatty Acid, Dicarboxylate | hexadecenedioate (C16:1-DC)* | 0.123 | 2.491 | 0.013 |
| Lysine Metabolism | N6-methyllysine | 0.106 | 2.370 | 0.018 |
| Unknown metabolite | X-15461 | -0.131 | -2.768 | 0.006 |
| Food Component/Plant | Ergothioneine | 0.121 | 2.526 | 0.012 |

Adjusted R^2^ = 0.145, p < 0.001.
