# Supplementary material for: Biomarkers of oxidative stress, diet and exercise distinguish soldiers selected and non-selected for special forces training
Source: Metabolomics. 2023 Apr 11;19(4):39. doi: 10.1007/s11306-023-01998-9 (PMC10090007; doi:10.1007/s11306-023-01998-9)
Supplement: Supplementary file 5 — Supplementary material 5 (DOCX 20.9 kb) [file 11306_2023_1998_MOESM5_ESM.docx]

## Supplemental Digital Content 4: Multiple Linear Regression Coefficients for Army Physical Fitness Scores

| **Subpathway** | **Name** | **β** | **t** | ***p*** |
| --- | --- | --- | --- | --- |
|  | Constant |  | 337.330 | 0.000 |
| Unknown Metabolite | X-11315 | 0.110 | 3.003 | 0.003 |
| Fatty Acid, Dicarboxylate | 3-carboxy-4-methyl-5-propyl-2-furanpropanoate (CMPF) | 0.120 | 3.363 | 0.001 |
| Fatty Acid Metabolism (Acylcarnitine) | arachidonoylcarnitine (C20:4) | 0.101 | 2.888 | 0.004 |
| Dihydrosphingomyelins | sphingomyelin (d18:0/20:0, d16:0/22:0)* | -0.095 | -2.678 | 0.008 |
| Lysine Metabolism | glutarylcarnitine (C5-DC) | 0.107 | 3.114 | 0.002 |
| Food Component/Plant | gluconate | 0.101 | 2.892 | 0.004 |
| Fatty Acid, Monohydroxy | 2-hydroxypalmitate | -0.256 | -3.740 | 0.000 |
| Long Chain Fatty Acid | eicosenoate (20:1) | 0.260 | 3.135 | 0.002 |
| Unknown metabolite | X-21736 | -0.106 | -2.789 | 0.005 |
| Unknown metabolite | X-25422 | 0.107 | 2.984 | 0.003 |
| Unknown metabolite | X-22162 | -0.108 | -2.931 | 0.003 |
| Unknown metabolite | X-21258 | 0.092 | 2.686 | 0.007 |
| Glycerolipid | Glycerol | -0.139 | -2.524 | 0.012 |
| Long Chain Fatty Acid | myristate (14:0) | 0.306 | 3.484 | 0.001 |
| Unknown metabolite | X-12844 | -0.082 | -2.346 | 0.019 |
| Long Chain Fatty Acid | palmitate (16:0) | -0.464 | -2.859 | 0.004 |
| Long Chain Fatty Acid | stearate (18:0) | 0.228 | 2.070 | 0.039 |

Adjusted R^2^ = 0.156, p < 0.001.
