# Supplementary material for: Biomarkers of oxidative stress, diet and exercise distinguish soldiers selected and non-selected for special forces training
Source: Metabolomics. 2023 Apr 11;19(4):39. doi: 10.1007/s11306-023-01998-9 (PMC10090007; doi:10.1007/s11306-023-01998-9)
Supplement: Supplementary file 6 — Supplementary material 6 (DOCX 36.0 kb) [file 11306_2023_1998_MOESM6_ESM.docx]

## Supplemental Digital Content 2: Metabolomic differences between selected and not selected candidates at pre-SFAS.

| **Superpathway** | **Subpathway** | **Name** | **Not Selected  (log_10_ auto-scaled AUC)** | **Selected  (log_10_ auto-scaled AUC)** | **p-value** | **FDR** |
| --- | --- | --- | --- | --- | --- | --- |
| Amino Acid | Glutathione Metabolism | cysteinylglycine | 0.098 | -0.07 | 0.001 | 0.016 |
| Amino Acid | Glutathione Metabolism | 4-hydroxy-nonenal-glutathione | 0.135 | 0.038 | 0.006 | 0.040 |
| Amino Acid | Glutathione Metabolism | 2-hydroxybutyrate/2-hydroxyisobutyrate | 0.071 | -0.182 | 0.008 | 0.046 |
| Amino Acid | Leucine, Isoleucine and Valine Metabolism | 3-hydroxyisobutyrate | -0.073 | -0.224 | 0.006 | 0.040 |
| Amino Acid | Leucine, Isoleucine and Valine Metabolism | alpha-hydroxyisocaproate | 0.069 | -0.226 | 0.008 | 0.045 |
| Amino Acid | Leucine, Isoleucine and Valine Metabolism | 1-carboxyethylleucine | 0.286 | 0.157 | 0.000 | 0.005 |
| Amino Acid | Leucine, Isoleucine and Valine Metabolism | 1-carboxyethylvaline | 0.105 | -0.023 | 0.002 | 0.020 |
| Amino Acid | Lysine Metabolism | N6-methyllysine | -0.331 | -0.111 | 0.007 | 0.042 |
| Amino Acid | Lysine Metabolism | glutarylcarnitine (C5-DC) | 0.175 | 0.291 | 0.005 | 0.034 |
| Amino Acid | Lysine Metabolism | pipecolate | -0.271 | -0.009 | 0.001 | 0.015 |
| Amino Acid | Methionine, Cysteine, SAM and Taurine Metabolism | N-acetylmethionine | 0.031 | -0.141 | 0.005 | 0.036 |
| Amino Acid | Methionine, Cysteine, SAM and Taurine Metabolism | N-acetylmethionine sulfoxide | 0.131 | -0.017 | 0.003 | 0.028 |
| Amino Acid | Phenylalanine Metabolism | 1-carboxyethylphenylalanine | 0.094 | -0.098 | 0.000 | 0.008 |
| Amino Acid | Tyrosine Metabolism | 3-methoxytyrosine | 0.071 | -0.169 | 0.002 | 0.020 |
| Carbohydrate | Glycolysis, Gluconeogenesis, and Pyruvate Metabolism | lactate | 0.112 | -0.265 | 0.000 | 0.001 |
| Carbohydrate | Glycolysis, Gluconeogenesis, and Pyruvate Metabolism | pyruvate | 0.111 | -0.12 | 0.001 | 0.010 |
| Carbohydrate | Pentose Metabolism | arabonate/xylonate | -0.109 | 0.143 | 0.000 | 0.009 |
| Carbohydrate | Pentose Metabolism | lyxonate | 0.075 | 0.25 | 0.002 | 0.024 |
| Cofactors and Vitamins | Nicotinate and Nicotinamide Metabolism | trigonelline (N'-methylnicotinate) | -0.063 | 0.356 | 0.000 | 0.001 |
| Lipid | Corticosteroids | cortisone | 0.203 | 0.293 | 0.004 | 0.030 |
| Lipid | Dihydrosphingomyelins | sphingomyelin (d18:0/18:0, d19:0/17:0)* | 0.061 | -0.18 | 0.000 | 0.007 |
| Lipid | Dihydrosphingomyelins | sphingomyelin (d18:0/20:0, d16:0/22:0)* | 0.082 | -0.159 | 0.000 | 0.007 |
| Lipid | Endocannabinoid | N-stearoyltaurine | 0.181 | 0.039 | 0.001 | 0.013 |
| Lipid | Endocannabinoid | oleoyl ethanolamide | 0.151 | -0.008 | 0.001 | 0.010 |
| Lipid | Fatty Acid Metabolism (Acyl Carnitine, Dicarboxylate) | adipoylcarnitine (C6-DC) | 0.362 | 0.198 | 0.000 | 0.005 |
| Lipid | Fatty Acid Metabolism (Acyl Carnitine, Dicarboxylate) | octadecanedioylcarnitine (C18-DC)* | 0.358 | 0.215 | 0.002 | 0.020 |
| Lipid | Fatty Acid Metabolism (Acyl Carnitine, Dicarboxylate) | octadecenedioylcarnitine (C18:1-DC)* | 0.064 | -0.177 | 0.000 | 0.006 |
| Lipid | Fatty Acid Metabolism (Acyl Carnitine, Medium Chain) | laurylcarnitine (C12) | 0.029 | -0.173 | 0.001 | 0.010 |
| Lipid | Fatty Acid Metabolism (Acyl Carnitine, Medium Chain) | hexanoylcarnitine (C6) | 0.041 | -0.218 | 0.002 | 0.020 |
| Lipid | Fatty Acid Metabolism (Acyl Carnitine, Medium Chain) | decanoylcarnitine (C10) | 0.035 | -0.177 | 0.003 | 0.028 |
| Lipid | Fatty Acid Metabolism (Acyl Carnitine, Monounsaturated) | 5-dodecenoylcarnitine (C12:1) | 0.192 | 0.042 | 0.008 | 0.045 |
| Lipid | Fatty Acid Metabolism (Acyl Carnitine, Monounsaturated) | myristoleoylcarnitine (C14:1)* | 0.07 | -0.208 | 0.000 | 0.005 |
| Lipid | Fatty Acid Metabolism (Acyl Carnitine, Polyunsaturated) | arachidonoylcarnitine (C20:4) | -0.012 | 0.131 | 0.009 | 0.050 |
| Lipid | Fatty Acid, Dicarboxylate | 3-carboxy-4-methyl-5-propyl-2-furanpropanoate | -0.059 | 0.173 | 0.006 | 0.040 |
| Lipid | Fatty Acid, Dicarboxylate | octadecenedioate (C18:1-DC) | 0.279 | 0.119 | 0.000 | 0.005 |
| Lipid | Fatty Acid, Dicarboxylate | hexadecenedioate (C16:1-DC)* | 0.251 | 0.133 | 0.000 | 0.006 |
| Lipid | Fatty Acid, Dicarboxylate | hydroxy-3-carboxy-4-methyl-5-propyl-2-furanpropanoate* | -0.087 | 0.143 | 0.001 | 0.016 |
| Lipid | Fatty Acid, Dicarboxylate | octadecadienedioate (C18:2-DC)* | 0.155 | 0.028 | 0.002 | 0.023 |
| Lipid | Fatty Acid, Monohydroxy | 3-hydroxydecanoate | -0.042 | -0.104 | 0.003 | 0.027 |
| Lipid | Fatty Acid, Monohydroxy | 3-hydroxyoctanoate | 0.053 | -0.092 | 0.002 | 0.018 |
| Lipid | Fatty Acid, Monohydroxy | 3-hydroxylaurate | 0.035 | -0.163 | 0.005 | 0.034 |
| Lipid | Fatty Acid, Monohydroxy | 2-hydroxypalmitate | 0.051 | -0.202 | 0.006 | 0.040 |
| Lipid | Fatty Acid, Monohydroxy | 13-hydroxyoctadecadienoic acid (HODE) + 9-HODE | 0.017 | -0.236 | 0.006 | 0.040 |
| Lipid | Glycerolipid Metabolism | glycerol | 0.007 | -0.154 | 0.000 | 0.008 |
| Lipid | Glycerolipid Metabolism | glycerol 3-phosphate | -0.08 | -0.229 | 0.004 | 0.034 |
| Lipid | Ketone Bodies | 3-hydroxybutyrate | -0.073 | -0.446 | 0.000 | 0.009 |
| Lipid | Long Chain Monounsaturated Fatty Acid | 10-heptadecenoate (17:1n7) | 0.015 | -0.148 | 0.006 | 0.040 |
| Lipid | Long Chain Monounsaturated Fatty Acid | 10-nonadecenoate (19:1n9) | 0.093 | -0.107 | 0.002 | 0.020 |
| Lipid | Long Chain Monounsaturated Fatty Acid | eicosenoate (20:1) | 0.072 | -0.192 | 0.001 | 0.017 |
| Lipid | Long Chain Monounsaturated Fatty Acid | oleate/vaccenate (18:1) | 0.091 | -0.136 | 0.001 | 0.012 |
| Lipid | Long Chain Polyunsaturated Fatty Acid (n3 and n6) | linoleate (18:2n6) | 0.052 | -0.121 | 0.001 | 0.013 |
| Lipid | Long Chain Polyunsaturated Fatty Acid (n3 and n6) | docosadienoate (22:2n6) | 0.056 | -0.12 | 0.003 | 0.029 |
| Lipid | Long Chain Polyunsaturated Fatty Acid (n3 and n6) | tetradecadienoate (14:2)* | 0.05 | -0.188 | 0.003 | 0.024 |
| Lipid | Long Chain Polyunsaturated Fatty Acid (n3 and n6) | dihomo-linoleate (20:2n6) | 0.048 | -0.124 | 0.005 | 0.034 |
| Lipid | Long Chain Polyunsaturated Fatty Acid (n3 and n6) | linolenate [alpha or gamma; (18:3n3 or 6)] | 0.044 | -0.123 | 0.003 | 0.028 |
| Lipid | Long Chain Polyunsaturated Fatty Acid (n3 and n6) | adrenate (22:4n6) | 0.128 | -0.038 | 0.004 | 0.034 |
| Lipid | Long Chain Saturated Fatty Acid | palmitate (16:0) | 0.021 | -0.183 | 0.000 | 0.009 |
| Lipid | Long Chain Saturated Fatty Acid | stearate (18:0) | 0.024 | -0.209 | 0.006 | 0.040 |
| Lipid | Long Chain Saturated Fatty Acid | myristate (14:0) | -0.024 | -0.148 | 0.006 | 0.040 |
| Lipid | Lysophospholipid | 1-linoleoyl-GPI (18:2)* | 0.12 | -0.223 | 0.000 | 0.001 |
| Lipid | Lysophospholipid | 1-arachidonoyl-GPI (20:4)* | 0.144 | -0.27 | 0.000 | 0.002 |
| Lipid | Medium Chain Fatty Acid | cis-4-decenoate (10:1n6)* | 0.087 | -0.109 | 0.004 | 0.030 |
| Lipid | Medium Chain Fatty Acid | 5-dodecenoate (12:1n7) | 0.127 | -0.024 | 0.007 | 0.044 |
| Lipid | Medium Chain Fatty Acid | (2 or 3)-decenoate (10:1n7 or n8) | 0.179 | 0.003 | 0.003 | 0.025 |
| Lipid | Phosphatidic acid | 1-palmitoyl-2-oleoyl-GPA (16:0/18:1) | 0.176 | -0.01 | 0.003 | 0.028 |
| Lipid | Pregnenolone Steroids | pregnenediol disulfate (C21H34O8S2)* | 0.11 | -0.151 | 0.007 | 0.041 |
| Lipid | Pregnenolone Steroids | pregnenetriol disulfate* | 0.077 | -0.222 | 0.000 | 0.009 |
| Lipid | Primary Bile Acid Metabolism | glycocholate | 0.066 | -0.166 | 0.008 | 0.046 |
| Lipid | Primary Bile Acid Metabolism | glycochenodeoxycholate | 0.156 | -0.123 | 0.000 | 0.009 |
| Lipid | Primary Bile Acid Metabolism | taurochenodeoxycholate | 0.227 | -0.011 | 0.000 | 0.007 |
| Lipid | Primary Bile Acid Metabolism | glycochenodeoxycholate 3-sulfate | 0.132 | 0.013 | 0.002 | 0.024 |
| Lipid | Progestin Steroids | 5alpha-pregnan-3beta,20alpha-diol disulfate | 0.118 | -0.078 | 0.003 | 0.024 |
| Lipid | Secondary Bile Acid Metabolism | glycoursodeoxycholate | 0.126 | -0.033 | 0.000 | 0.007 |
| Lipid | Secondary Bile Acid Metabolism | glycocholenate sulfate* | 0.113 | -0.221 | 0.000 | 0.002 |
| Lipid | Secondary Bile Acid Metabolism | taurocholenate sulfate* | 0.082 | -0.203 | 0.000 | 0.007 |
| Lipid | Sphingomyelins | sphingomyelin (d18:1/20:2, d18:2/20:1, d16:1/22:2)* | 0.227 | 0.097 | 0.000 | 0.006 |
| Lipid | Sterol | 3beta-hydroxy-5-cholestenoate | 0.089 | -0.157 | 0.006 | 0.039 |
| Partially Characterized Molecules | Partially Characterized Molecules | pentose acid* | -0.043 | 0.154 | 0.004 | 0.032 |
| Partially Characterized Molecules | Partially Characterized Molecules | bilirubin degradation product, C16H18N2O5 (3)** | -0.008 | -0.165 | 0.001 | 0.017 |
| Unknown Compound | Unknown Compound | X-11315 | -0.066 | 0.225 | 0.000 | 0.006 |
| Unknown Compound | Unknown Compound | X-11880 | 0.079 | -0.171 | 0.000 | 0.006 |
| Unknown Compound | Unknown Compound | X-12216 | 0.024 | 0.195 | 0.001 | 0.012 |
| Unknown Compound | Unknown Compound | X-12844 | 0.022 | 0.232 | 0.004 | 0.034 |
| Unknown Compound | Unknown Compound | X-13729 | 0.065 | 0.267 | 0.003 | 0.024 |
| Unknown Compound | Unknown Compound | X-15461 | -0.006 | 0.285 | 0.001 | 0.013 |
| Unknown Compound | Unknown Compound | X-16935 | 0.189 | 0.114 | 0.005 | 0.036 |
| Unknown Compound | Unknown Compound | X-18901 | 0.218 | 0.338 | 0.002 | 0.022 |
| Unknown Compound | Unknown Compound | X-18913 | 0.079 | -0.172 | 0.007 | 0.041 |
| Unknown Compound | Unknown Compound | X-21258 | -0.103 | 0.202 | 0.004 | 0.034 |
| Unknown Compound | Unknown Compound | X-21353 | 0.023 | -0.119 | 0.005 | 0.037 |
| Unknown Compound | Unknown Compound | X-21470 | 0.149 | -0.002 | 0.005 | 0.036 |
| Unknown Compound | Unknown Compound | X-21736 | 0.243 | 0.033 | 0.000 | 0.002 |
| Unknown Compound | Unknown Compound | X-22162 | -0.058 | 0.207 | 0.000 | 0.005 |
| Unknown Compound | Unknown Compound | X-23665 | 0.311 | 0.204 | 0.001 | 0.017 |
| Unknown Compound | Unknown Compound | X-24431 | 0.298 | 0.413 | 0.007 | 0.043 |
| Unknown Compound | Unknown Compound | X-25271 | 0.042 | 0.337 | 0.001 | 0.010 |
| Unknown Compound | Unknown Compound | X-25422 | -0.061 | 0.219 | 0.000 | 0.009 |
| Unknown Compound | Unknown Compound | X-25790 | 0.036 | 0.328 | 0.001 | 0.011 |
| Xenobiotics | Benzoate Metabolism | hippurate | 0.02 | 0.248 | 0.006 | 0.040 |
| Xenobiotics | Benzoate Metabolism | guaiacol sulfate | 0.031 | 0.25 | 0.001 | 0.013 |
| Xenobiotics | Benzoate Metabolism | 3-hydroxyhippurate | 0.005 | 0.232 | 0.001 | 0.011 |
| Xenobiotics | Benzoate Metabolism | catechol sulfate | 0.034 | 0.304 | 0.000 | 0.006 |
| Xenobiotics | Chemical | ectoine | 0.207 | 0.364 | 0.006 | 0.040 |
| Xenobiotics | Food Component/Plant | quinate | 0.16 | 0.442 | 0.000 | 0.007 |
| Xenobiotics | Food Component/Plant | S-allylcysteine | 0.129 | 0.276 | 0.008 | 0.046 |
| Xenobiotics | Food Component/Plant | ergothioneine | -0.012 | 0.207 | 0.000 | 0.009 |
| Xenobiotics | Food Component/Plant | gluconate | -0.095 | 0.047 | 0.000 | 0.006 |
| Xenobiotics | Food Component/Plant | N-acetylalliin | 0.177 | 0.359 | 0.002 | 0.022 |
| Values represent median log_10_ auto-scaled AUC. * Compounds that have not been officially confirmed based on a standard, but are identified with high confidence, FDR represents False Discovery Rate, C indicates Carbon, DC indicates Dicarboxylate | | | | | | |
