# Supplementary material for: Biomarkers of oxidative stress, diet and exercise distinguish soldiers selected and non-selected for special forces training
Source: Metabolomics. 2023 Apr 11;19(4):39. doi: 10.1007/s11306-023-01998-9 (PMC10090007; doi:10.1007/s11306-023-01998-9)

## Supplemental Digital Content 3: Metabolomic differences between selected and non-selected candidates at pre-SFAS. Values represent log_10_ auto-scaled AUC.


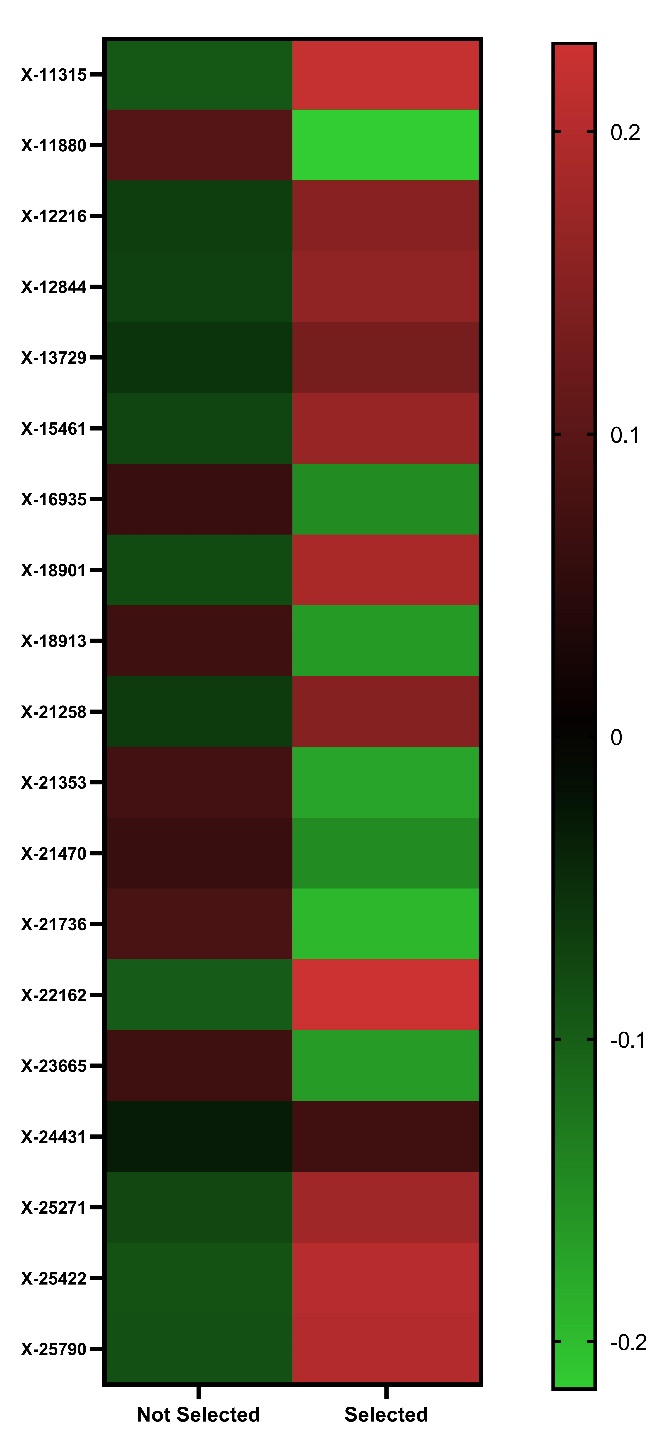

Supplement: Supplementary file 7 — Supplementary material 7 (DOCX 125.1 kb) [file 11306_2023_1998_MOESM7_ESM.docx]
