# Supplementary material for: Biomarkers of oxidative stress, diet and exercise distinguish soldiers selected and non-selected for special forces training
Source: Metabolomics. 2023 Apr 11;19(4):39. doi: 10.1007/s11306-023-01998-9 (PMC10090007; doi:10.1007/s11306-023-01998-9)
Supplement: Supplementary file 8 — Supplementary material 8 (DOCX 20.5 kb) [file 11306_2023_1998_MOESM8_ESM.docx]

## Supplemental Digital Content 8: Multiple Linear Regression Coefficients for Run 1

| **Subpathway** | **Name** | **β** | **t** | ***p*** |
| --- | --- | --- | --- | --- |
|  | (Constant) |  | 388.400 | 0.000 |
| Unknown metabolite | X-11315 | -0.135 | -3.626 | 0.000 |
| Glycerolipid | Glycerol | 0.147 | 2.927 | 0.004 |
| Pentose metabolism | arabonate/xylonate | -0.115 | -3.079 | 0.002 |
| Glutathione Metabolism | 4-hydroxy-nonenal-glutathione | -0.154 | -4.165 | 0.000 |
| Unknown metabolite | X-23665 | 0.132 | 3.251 | 0.001 |
| Medium Chain Fatty Acid | laurate (12:0) | -0.119 | -2.679 | 0.008 |
| Unknown metabolite | X-25422 | -0.087 | -2.366 | 0.018 |
| Leucine, Isoleucine and Valine Metabolism | alpha-hydroxyisocaproate | 0.089 | 2.342 | 0.020 |
| Fatty Acid Metabolism (Acylcarnitine) | 5-dodecenoylcarnitine (C12:1) | 0.138 | 3.195 | 0.001 |
| Ketone Body | 3-hydroxybutyrate (BHBA) | -0.141 | -2.758 | 0.006 |
| Dihydrosphingomyelins | sphingomyelin (d18:0/18:0, d19:0/17:0)* | 0.095 | 2.550 | 0.011 |
| Fatty Acid Metabolism (Acylcarnitine) | arachidonoylcarnitine (C20:4) | -0.092 | -2.460 | 0.014 |
| Leucine, Isoleucine and Valine Metabolism | 1-carboxyethylleucine | 0.077 | 2.037 | 0.042 |

Adjusted R^2^ = 0.160, p < 0.001.
