# Supplementary material for: Biomarkers of oxidative stress, diet and exercise distinguish soldiers selected and non-selected for special forces training
Source: Metabolomics. 2023 Apr 11;19(4):39. doi: 10.1007/s11306-023-01998-9 (PMC10090007; doi:10.1007/s11306-023-01998-9)
Supplement: Supplementary file 9 — Supplementary material 9 (DOCX 20.5 kb) [file 11306_2023_1998_MOESM9_ESM.docx]

## Supplemental Digital Content 7: Multiple Linear Regression Coefficients for Road March 2

| **Subpathway** | **Name** | **β** | **t** | ***p*** |
| --- | --- | --- | --- | --- |
|  | (Constant) |  | 204.339 | 0.000 |
| Unknown metabolite | X-11315 | -0.143 | -3.790 | 0.000 |
| Glycerolipid | Glycerol | 0.102 | 2.287 | 0.023 |
| Phenylalanine Metabolism | 1-carboxyethylphenylalanine | 0.160 | 4.350 | 0.000 |
| Unknown metabolite | X-25422 | -0.109 | -2.890 | 0.004 |
| Unknown metabolite | X-23665 | 0.138 | 3.604 | 0.000 |
| Unknown metabolite | X-21258 | -0.108 | -2.929 | 0.004 |
| Dihydrosphingomyelins | sphingomyelin (d18:0/18:0, d19:0/17:0)* | 0.092 | 2.466 | 0.014 |
| Food Component/Plant | S-allylcysteine | -0.082 | -2.178 | 0.030 |
| Fatty Acid Metabolism (Acylcarnitine) | hexanoylcarnitine (C6) | 0.115 | 2.619 | 0.009 |
| Unknown metabolite | X-22162 | -0.095 | -2.461 | 0.014 |
| Fatty Acid, Monohydroxy | 3-hydroxydecanoate | -0.110 | -2.430 | 0.015 |
| Unknown metabolite | X-11880 | 0.095 | 2.405 | 0.016 |
| Primary Bile Acid Metabolism | glycocholate | -0.076 | -2.022 | 0.044 |

Adjusted R^2^ = 0.202, p < 0.001.
