# Supplementary material for: Biomarkers of oxidative stress, diet and exercise distinguish soldiers selected and non-selected for special forces training
Source: Metabolomics. 2023 Apr 11;19(4):39. doi: 10.1007/s11306-023-01998-9 (PMC10090007; doi:10.1007/s11306-023-01998-9)
Supplement: Supplementary file 10 — Supplementary material 10 (DOCX 18.7 kb) [file 11306_2023_1998_MOESM10_ESM.docx]

| **Chromatographic Conditions** | **UPLC-MS/MS** | **UPLC-MS/MS** | **UPLC-MS/MS** | **HILIC/UPLC-MS/MS** |
| --- | --- | --- | --- | --- |
| ESI | Positive | Positive | Negative | Negative |
| Chromatographical optimization | Hydrophobic (polar) | Hydrophilic (lipid) |  |  |
| Column Size | C18 (1.7um 2.1 x 100mm) | C18 (1.7um 2.1 x 100mm) | C18 (1.7um 2.1 x 100mm) | C18 (1.7um 2.1 x 100mm) |
| Mobile Phase A | 0.1% formic acid and 0.05% PFPA in water, pH ~2.5 | 0.1% formic acid and 0.05% PFPA in water, pH ~2.5 | 6.5 mM ammonium bicarbonate in water, pH 8 | 10 mM ammonium formate in 15% water/ 5% methanol/ 80% acetonitrile (effective pH 10.16 with NH4OH) |
| Mobile Phase B | 0.1% formic acid and 0.05% PFPA in methanol, pH ~2.5 | 0.1% formic acid and 0.05% PFPA in 50% methanol/ 50% acetonitrile, pH ~2.5 | 6.5 mM ammonium bicarbonate in 95% methanol/ 5% water | 10 mM ammonium formate in 50% water/ 50% acetonitrile (effective pH 10.60 with NH4OH) |
| Flow rate (ml/min) | 0.35 | 60 | 0.35 | 0.5 |
| Gradient Method | Linear gradient (5% to 80% B over 3.35 min) | Linear gradient (40% to 99.5% over 1.0 min, hold 99.5% B for 2.4 min) | Linear gradient (0.5 to 70% over 4.0 min, then rapid gradient to 99% B in 0.5 min) | Linear gradient (5% to 50% B in 3.5 min, then linear gradient from 50 to 90% B in 2 min) |
| Spray Voltage (V) | 4000 | 4200 | 3300 | 3000 |
| Mass range (m/z) | 70-1000 | 110-1000 | 80-1000 | 80-1000 |
| Sheath Gas (au) | 70 | 35 | 70 | 60 |
| Auxiliary Gas (au) | 35 | 35 | 15 | 20 |
| Source Temp (°C) | 300 | 400 | 300 | 300 |
| Ion Transfer Tube Temp. (°C) | 250 | 320 | 250 | 250 |
| Norm. Collision Energy (au) | 52, 65, 78 | 52, 65, 78 | 52, 65, 78 | 48, 60, 72 |
| MS AGC target (au) | 1E+6 | | | |
| MS Max Fill Time (ms) | 60 | | | |
| MSn Ion Target (au) | 2E+5 | | | |
| MSn Max Fill Time (ms) | 120 | | | |
| MSn Isolation Window (m/z) | 120 | | | |
| MSn Dynamic Exclusion Time (s) | 3 | | | |
| S-Lens RF Level | 3 | | | |
| MSn Ion Target (au) | 40 | 50 | 40 | 25 |

Supplement 1: UPLC-MS Methodology
